# Internet healthcare in Chinese public hospitals: Towards high-quality development (1986-present)

**DOI:** 10.3934/publichealth.2026032

**Published:** 2026-05-19

**Authors:** Dan Du, Guodong Sun, Lili Yuan, Yiyun Yao, Tong Yang, Junliang Gao, Lei Zhang

**Affiliations:** 1 Department of Medical Affairs, Lanzhou University First Hospital, Lanzhou 730000, China; 2 General Surgery Department, Lanzhou University First Hospital, Lanzhou 730000, China

**Keywords:** China, internet healthcare, public hospitals, telemedicine, digital health, health policy, equity

## Abstract

**Background:**

Internet healthcare has become a key part of China's hospital-centered health system. Driven by “Internet Plus Healthcare”, Healthy China 2030, and public-hospital high-quality development policies, it has evolved from remote consultation experiments into regulated online-offline care pathways. This review traces its development from the first documented remote medical practice in 1986 to the present, focusing on policy, institutional models, clinical evidence, governance challenges, and reform.

**Methods:**

We conducted a structured narrative review of English- and Chinese-language sources on internet healthcare in Chinese public hospitals. PubMed/MEDLINE, Web of Science Core Collection, China National Knowledge Infrastructure (CNKI), and official policy sources were searched. Eligible sources addressed internet hospitals, telemedicine, online follow-ups, remote monitoring, e-prescriptions, insurance payments, digital governance, clinical outcomes, patient safety, equity, or implementation barriers in mainland China.

**Results:**

Internet healthcare progressed through early telemedicine, institutional network expansion, internet-hospital development, and pandemic-driven normalization. The 2018 regulatory framework positioned internet hospitals as extensions of licensed physical medical institutions, thereby permitting online follow-ups for common and chronic diseases while preserving offline accountability. During COVID-19, online consultation, e-prescriptions, drug delivery, and insurance payments rapidly expanded. Evidence suggests benefits for chronic disease management, medication adherence, cardiovascular secondary prevention, and reduced travel burden. However, evidence remains limited for diagnostic accuracy, adverse events, emergency escalation, and long-term outcomes. Persistent barriers include quality variation, workload, cybersecurity, data fragmentation, artificial intelligence (AI) accountability, reimbursement design, regional inequity, and digital exclusion among older adults.

**Conclusion:**

China's model may be understood as a hospital-centered extension of public-hospital functions rather than a stand-alone virtual-care system. Future development should prioritize outcome-based evaluations, safety governance, equitable access, data interoperability, and accountability for internet-based and AI-assisted care.

## 1. Introduction

### 1.1. Background and rationale

Over the past four decades, China has increasingly used internet-enabled healthcare to improve access, efficiency, and coordination within its hospital-centered health system. Internet healthcare broadly refers to the delivery or support of medical services through digital and internet technologies, including online consultation, inter-institutional telemedicine, remote monitoring, mobile health applications, and related “Internet Plus Healthcare” services. In China, these services have been promoted as responses to long-standing structural challenges, including the uneven distribution of high-quality medical resources, limited access in rural and remote areas, and overcrowding in tertiary hospitals. As the backbone of China's healthcare delivery system, public hospitals have played a central role in this transformation by integrating online platforms with conventional in-person care and extending specialist services beyond hospital walls [1],[2].

### 1.2. Scope of internet healthcare covered in this review

In this review, internet healthcare is understood as a broad service and governance framework rather than a single technology or platform. It includes patient-facing services, such as online consultations, online follow-ups for selected common and chronic diseases, e-prescriptions, drug-delivery coordination, appointment services, and health education. Additionally, it includes provider-to-provider and institution-to-institution services, such as remote consultations, remote imaging and pathology support, tele-intensive care unit (ICU) collaborations, emergency guidance, multidisciplinary discussions, and cross-regional referrals. In addition, internet healthcare covers longitudinal care functions, including remote monitoring, chronic disease management, and post-discharge follow-ups, as well as platform-level governance functions such as online insurance settlements, electronic medical record integration, data governance, cybersecurity, and emerging artificial intelligence (AI)-assisted triage [3].

### 1.3. Definition of high-quality development

In this article, “high-quality development” is used as a policy-grounded concept in China's public hospital reform rather than as a general expression of service improvement. The 2021 Opinion on Promoting the High-Quality Development of Public Hospitals emphasized that public hospitals should shift from scale expansion to quality and efficiency, from extensive management to refined management, and from resource input-driven growth to development supported by talent, technology, and institutional innovation [4]. Applied to internet healthcare, high-quality development means more than increasing the number of internet hospitals or online visits. It refers to digitally enabled services that improve clinical quality, patient safety, efficiency, equity, continuity of care, patient experience, data governance, and public-health resilience while maintaining the public-interest mission of public hospitals.

Additionally, this requires a balanced assessment of benefits and risks. Internet healthcare may improve access, reduce travel burden, and support chronic disease follow-up, but it may also create challenges related to diagnostic accuracy, prescription safety, digital exclusion, physician workload, and fragmented data systems. Therefore, this review evaluates internet healthcare not only by platform expansion, but also by its implications for quality, safety, equity, continuity, and governance [5]–[7].

## 2. Materials and methods

### 2.1. Review design

This article was conducted as a structured narrative review of internet healthcare in Chinese public hospitals. The review aimed to synthesize historical, policy, institutional, clinical, and implementation evidence rather than to perform quantitative pooling, a formal risk-of-bias assessment, or a meta-analysis. Because the topic spans long-term policy evolution, regulatory development, hospital practice, and emerging clinical evidence, we included peer-reviewed studies, official policy documents, and selected institutional sources.

### 2.2. Data sources and search strategy

We identified English- and Chinese-language sources relevant to internet healthcare in Chinese public hospitals from January 1986 to October 2025. English-language literature was searched in PubMed/MEDLINE and the Web of Science Core Collection, and Chinese-language literature was searched in China National Knowledge Infrastructure (CNKI). To identify policy and regulatory documents, we also searched official websites of the State Council of the People's Republic of China, the National Health Commission, the National Healthcare Security Administration, provincial health commissions, and representative public hospitals.

Search concepts combined terms related to internet-based healthcare, China, and hospital-based care. English terms included “internet hospital”, “internet healthcare”, “Internet Plus Healthcare”, telemedicine, telehealth, online consultations, remote consultations, remote monitoring, digital health, mobile health, China, public hospitals, tertiary hospitals, county hospitals, medical consortiums, and medical alliances. Equivalent Chinese terms were used for CNKI searches, including terms for internet hospitals, internet healthcare, remote consultations, online follow-ups, smart hospitals, public hospitals, tertiary hospitals, county hospitals, medical alliances, and county medical communities.

### 2.3. Eligibility criteria, source selection, and information extraction

Sources were considered eligible if they focused on mainland China and addressed internet hospitals, internet diagnoses and treatments, telemedicine, online follow-ups, remote consultations, remote monitoring, e-prescriptions, medical-insurance payments, digital health governance, or related services. We prioritized sources that provided evidence relevant to public hospitals, including policy evolution, regulatory frameworks, institutional models, service pathways, clinical outcomes, patient safety, access, equity, workforce, data governance, or implementation barriers.

Sources were excluded if they were outside the scope of mainland China's healthcare delivery system, were not directly related to internet healthcare or public-hospital practice, lacked accessible full text, or did not provide verifiable policy, historical, institutional, or empirical information. Candidate sources were reviewed for relevance to the narrative structure of the manuscript, and uncertainties regarding inclusion or interpretation were resolved through discussion among the author team.

Evidence was narratively synthesized according to the revised structure of the manuscript: historical evolution, policy and regulatory development, institutional practice, clinical outcomes and patient safety, implementation challenges, and future directions.

### 2.4. Evidence synthesis and citation hierarchy

To improve transparency and reduce overreliance on non-peer-reviewed sources, we applied a source hierarchy. Peer-reviewed empirical studies were prioritized for claims about clinical effectiveness, diagnostic accuracy, patient safety, patient experience, and service quality. Official policy documents were used to support regulatory milestones, service definitions, reimbursement policies, and public-hospital reform concepts. Chinese-language academic and policy sources were used when they provided essential information on China-specific institutions, such as medical alliances, county medical communities, internet hospital regulation, and public-hospital high-quality development.

## 3. Historical origins and evolution of internet healthcare in China

The development of internet healthcare in Chinese public hospitals can be divided into four overlapping phases: early telemedicine experimentation, institutional network expansion, the emergence of internet hospitals, and pandemic-driven normalization.

### 3.1. Early telemedicine initiatives and foundational experiments, 1986–2000

China's earliest documented remote medical practice can be traced to 1986, when emergency consultations were reportedly conducted through radiotelegraphy for ship crews, representing an early form of long-distance medical assistance before widespread internet access [8]. In 1988, the PLA General Hospital conducted a satellite-enabled consultation with a German hospital for a neurosurgical case, which has often been regarded as the formal starting point of modern telemedicine in China [9],[10]. During the 1990s, telemedicine in China remained largely experimental and mainly relied on satellite links, telephone lines, integrated services digital network (ISDN), and videoconferencing rather than web-based platforms [11],[12].

In 1995, Shanghai launched an early institutional telemedicine pilot project and established a dedicated telemedicine center, thus reflecting the first wave of hospital-led experimentation [13]. In 1997, the Jin-Wei Telemedicine Network was launched to connect hospitals across multiple provinces through satellite-based communication, thus laying an institutional foundation for later inter-hospital teleconsultation networks [14]. By the end of the 1990s, China moved from scattered telemedicine experiments toward organized institutional networks, although services were still mainly provider-to-provider rather than patient-facing [12].

### 3.2. Network expansion and institutional telemedicine, 2001–2014

The early 2000s marked a transition from fragmented pilots to broader institutional telemedicine deployment. In 2001, the Junwei II Telemedicine Network expanded to approximately 300 hospitals nationwide and supported remote consultation and medical education services [12]. During this period, several provinces, including western and border regions, developed regional telemedicine platforms to connect tertiary hospitals with lower-level institutions [15]. In 2010, national investment in telemedicine infrastructure supported network development across central and western provinces, thereby linking tertiary hospitals with county-level hospitals to improve access to specialist expertise; by 2013, more than 2000 healthcare institutions were reported to be providing telemedicine services, thus suggesting that telemedicine had become an established institutional function rather than a small-scale pilot activity [15].

In 2014, the National Health and Family Planning Commission issued the Opinion on Advancing Telemedical Services, formally positioning telemedicine as an inter-institutional medical activity [16]. Thus, by the mid-2010s, China developed a relatively mature institutional telemedicine infrastructure, though consumer-facing internet healthcare remained limited.

### 3.3. Emergence of internet hospitals and regulatory liberalization, 2015–2019

The mid-2010s marked the emergence of internet hospitals as a distinct organizational model within China's digital health system [17]. In 2015, the State Council's “Internet Plus” action plan promoted the integration of internet technologies with public services, including healthcare [18]. In 2016, the Healthy China 2030 Planning Outline further positioned health informatization and health-system modernization as part of China's long-term health strategy [19].

Wuzhen Internet Hospital, established by WeDoctor, became a widely cited early model that combined online consultations, e-prescriptions, chronic disease services, and pharmacy linkages [17]. Xie et al. identified 68 internet hospitals by March 2017, of which 43 had been put into use; however, they also found that many remained immature and faced limitations such as online physician scarcity and incomplete insurance coverage [1]. Lai et al. later identified 268 officially licensed internet hospitals and reported that public hospitals, especially tertiary hospitals, were important institutional actors in internet-hospital development [17].

In 2018, the State Council issued the Opinions on Promoting the Development of “Internet Plus Healthcare”, and the National Health Commission released trial measures on internet diagnoses and treatments, internet hospitals, and telemedicine services [2],[3]. These 2018 policies marked a regulatory turning point by allowing internet hospitals to develop under the responsibility of licensed physical medical institutions [3].

### 3.4. Pandemic-driven acceleration and present policy horizon, 2020–present

The COVID-19 pandemic accelerated the normalization of internet healthcare in China by creating an urgent need for remote triage, online follow-ups, e-prescriptions, and reduced in-person hospital visits. Wu et al. reported that mobile health technologies and internet hospitals helped alleviate the unavailability, inaccessibility, and inequity of health services during the COVID-19 outbreak in China [20]. Xu et al. documented 711 internet hospitals in mainland China by 16 July 2020, including 215 established in early 2020 as part of the emergency response to COVID-19 [21]. The same national cross-sectional study found that internet hospitals provided services such as online consultations, prescriptions, drug delivery, and medical-insurance-related functions during the pandemic [21]. In 2020, the National Healthcare Security Administration and the National Health Commission issued guidance that opened reimbursement channels for eligible “Internet Plus” medical insurance services, particularly online follow-up for common and chronic diseases [22].

**Figure 1.**
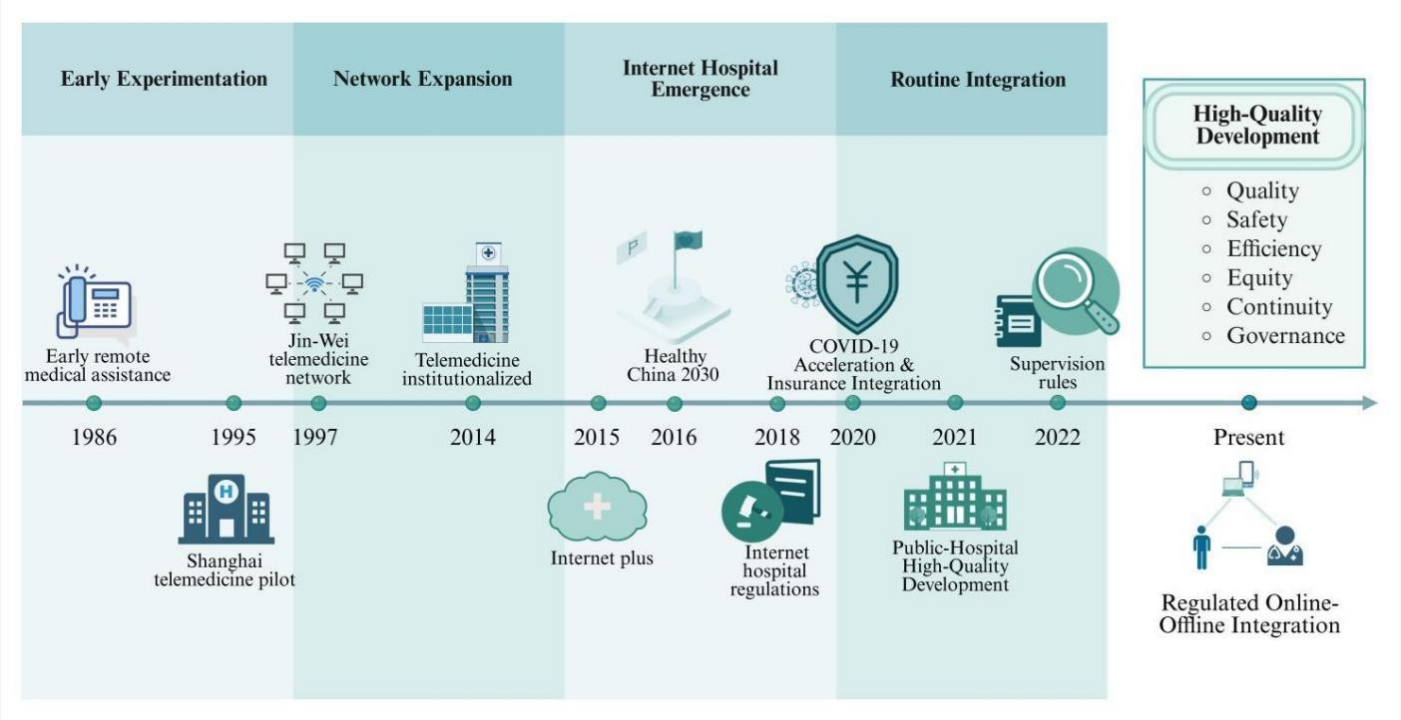
Development milestones of internet healthcare in Chinese public hospitals.

Since 2021, policy emphasis has shifted from emergency expansion toward routine integration, interoperability, quality governance, smart-hospital construction, and the high-quality development of public hospitals [4],[23]. Recent multicenter evidence from internet hospitals operated by physical hospitals suggests that online consultation services have become part of routine hospital operation, although service quality, workflow standardization, and outcome evaluation remain as continuing challenges [24]. The major historical phases and milestones of this evolution are summarized in Figure 1.

This timeline summarizes the major stages in the development of internet healthcare in Chinese public hospitals from the first documented remote medical practice in 1986 to the present. Key milestones include early remote medical assistance, regional telemedicine pilots, the Jin-Wei telemedicine network, institutionalization of telemedicine, the Internet Plus Action Plan, Healthy China 2030, internet hospital regulations, COVID-19-driven acceleration and insurance integration, public-hospital high-quality development, and subsequent supervision rules. The figure highlights the transition from early experimentation and network expansion to internet hospital emergence and regulated online-offline integration. Additionally, it summarizes the policy-oriented dimensions of high-quality development, including quality, safety, efficiency, equity, continuity, and governance.

## 4. Policy and regulatory landscape

Strong top-down policy support has been a decisive factor in the growth of internet healthcare in China. The Chinese government has framed “Internet Plus Healthcare” as a policy tool to optimize medical-resource allocation, thereby expanding access, improving service efficiency, and supporting broader health-system reform. This section consolidates the major policy and regulatory milestones that shaped internet healthcare in Chinese public hospitals, with particular attention to the service scope, institutional accountability, reimbursement, quality governance, and data security.

### 4.1. Institutionalizing telemedicine and restricting direct online diagnosis (2014)

The 2014 Opinion on Advancing Telemedical Services marked a pivotal regulatory moment by defining telemedicine as a medical service that should operate within the responsibility framework of licensed medical institutions. This policy reflected an early regulatory priority: remote care should only be permitted when medical quality, patient safety, institutional accountability, medical-record management, and professional responsibility could be ensured. Under this framework, individual physicians and non-medical commercial platforms were not permitted to independently provide direct-to-consumer online diagnoses outside the governance structure of licensed medical institutions [25].

Later, this restriction became widely interpreted as China's “online diagnosis ban,” although its practical function was not to prohibit all telemedicine activity but to anchor remote diagnosis within institution-based clinical governance. Consequently, early telemedicine in China mainly developed through business-to-business and hospital-to-hospital models, including remote consultations, remote imaging, remote pathology, professional training, and inter-hospital referral support [15].

### 4.2. From strategic encouragement to formal internet hospital regulation (2015–2018)

Between 2015 and 2018, China's internet healthcare policy shifted from broad strategic encouragement to more operational regulation. Early national strategies, including the “Internet Plus” initiative and Healthy China 2030, created policy momentum for digital health, smart services, and telemedicine, but they did not provide detailed rules for internet diagnoses, internet hospitals, or medical liability [17]. A major turning point occurred in 2018, when the State Council issued the Guidance on Promoting the Development of “Internet Plus Healthcare”. This document encouraged medical institutions to use internet technologies to optimize existing care pathways, expand online follow-ups, support chronic disease management, develop family-doctor contract services, promote e-prescriptions, explore online medical-insurance settlement, and cooperate with qualified third-party platforms under regulatory supervision [2].

Later in 2018, the National Health Commission and the National Administration of Traditional Chinese Medicine issued three trial measures on internet diagnoses and treatments, internet hospitals, and telemedicine services. These measures established the core regulatory basis for internet hospitals by requiring online diagnoses and treatments to rely on licensed physical medical institutions, thus limiting internet diagnoses and treatments mainly to online follow-ups for selected common and chronic diseases and “Internet Plus” family-doctor contract services, and allowing internet hospitals to operate either as the second name of a physical medical institution or as a platform established with institutional support and regulatory approval [3].

This policy design explains why Chinese internet hospitals differ from many stand-alone commercial telehealth platforms: they are expected to integrate online services with offline records, offline reassessments, institutional quality control, and physician accountability [17]. Xie et al. showed that early internet hospitals were still immature in 2017, with limited online physician availability and incomplete insurance coverage, underscoring why formal regulation and reimbursement policies became necessary for wider adoption [1]. Therefore, the 2018 policy package should be interpreted not as unrestricted liberalization, but as a controlled pathway for public hospitals and licensed institutions to expand internet healthcare under online-offline accountability [3].

### 4.3. Pandemic-era emergency policies and medical-insurance integration (2020)

The COVID-19 pandemic transformed internet healthcare from a regulated innovation into a key component of emergency service continuity. Wu et al. reported that mobile health technologies and internet hospitals helped alleviate service unavailability, inaccessibility, and inequity during the COVID-19 outbreak in China [20]. Xu et al. documented 711 internet hospitals in mainland China by 16 July 2020, including 215 established in early 2020 as part of the emergency response; the same study also found that many internet hospitals provided online consultations, prescriptions, drug delivery, and medical-insurance-related functions during the pandemic [21].

The most important financing breakthrough occurred in 2020, when the National Healthcare Security Administration and the National Health Commission issued guidance on “Internet Plus” medical-insurance services during COVID-19 prevention and control. This policy allowed eligible internet medical institutions to include online follow-ups for common and chronic diseases in basic medical-insurance payments, provided that the services were delivered by approved internet hospitals or institutions approved to conduct internet diagnoses and treatments. Additionally, it supported electronic prescriptions, offline drug dispensing through medical institutions or designated retail pharmacies, and online settlements of medical-insurance-covered consultation and drug costs. To protect insurance-fund safety and patient safety, the policy required real-name online visits, prescription review, and safeguards against fabricated medical services [22]. Thus, the COVID-19 period did not merely increase the online service volume; it accelerated the linkage between internet hospitals, chronic disease follow-ups, e-prescriptions, drug delivery, and medical-insurance payments [21].

### 4.4. Public-hospital high-quality development and smart-hospital governance (2021–present)

Since 2021, internet healthcare policies have been increasingly embedded in China's broader public-hospital high-quality development agenda. The 2021 Opinion on Promoting the High-Quality Development of Public Hospitals required public hospitals to shift from scale expansion to quality and efficiency, from extensive management to refined management, and from material-resource input toward talent- and technology-driven development. For internet healthcare, this policy orientation means that platform expansion alone is insufficient unless online services improve the quality, safety, efficiency, equity, continuity, and patient experience [4]. The subsequent Public Hospital High-Quality Development Promotion Action (2021–2025) translated this reform agenda into a time-limited action plan for the 14th Five-Year Plan period, thereby emphasizing smart hospitals, online-offline integrated medical services, service-quality improvements, patient-experience improvements, and refined hospital management. Therefore, the 2021–2025 action plan should be interpreted as a policy horizon for public-hospital reform rather than as evidence that all high-quality development goals were fully achieved by 2025 [23].

Recent empirical evidence suggests that online consultation services in internet hospitals operated by physical hospitals have become part of the routine hospital operation, but service quality, workflow standardization, and outcome evaluation remain important challenges [24]. Moreover, the healthcare providers' readiness remains relevant to policy implementation, as pre-pandemic survey evidence showed that provider familiarity with e-hospitals and prior online healthcare experiences were associated with readiness to work in e-hospitals [26]. Physician interview evidence further indicates that internet hospitals can improve access but also create challenges related to patient expectations, doctor-patient boundaries, communication, and informed consent [27]. In 2022, the Internet Diagnosis and Treatment Supervision Rules (Trial) reinforced this quality-governance orientation by requiring internet diagnoses and treatments to be incorporated into routine supervision and by strengthening the role of provincial-level regulatory platforms. Therefore, the current regulatory landscape combines development incentives with stronger supervision, thus reflecting a transition from rapid policy-driven expansion toward regulated high-quality integration [28].

### 4.5. Payment, reimbursement, and sustainability

Payment policy has been a decisive factor in determining whether internet healthcare can become a routine public-hospital service rather than a temporary innovation. Before reimbursement mechanisms became more established, early internet hospitals faced limited insurance coverage, which reduced patient incentives and constrained institutional sustainability [1]. The 2020 medical-insurance guidance created a national policy pathway to reimburse eligible online follow-up services for common and chronic diseases during COVID-19 prevention and control, thereby supporting the institutionalization of online follow-ups, e-prescriptions, drug dispensing, and online settlements within internet hospitals [22].

Real-world evidence from an internet hospital showed that online regular follow-ups reduced the patient travel time and transportation costs, thus suggesting that reimbursement-supported online follow-ups can improve access and reduce non-medical care burdens [5]. However, payment integration should not be evaluated only by service uptake, because internet hospital consultations may also increase the outpatient frequency and expenses in some contexts [29]. This finding suggests that payment policy should be linked to quality indicators, clinical appropriateness, and the avoidance of low-value online visits rather than simply encouraging a higher online consultation volume. Moreover, patient-side evidence from western China shows that perceived usefulness, perceived ease of use, trust, and service accessibility influence the willingness to adopt e-hospitals, thus indicating that reimbursement alone is not sufficient for equitable uptake [30]. Therefore, for public hospitals, sustainable internet healthcare requires a combined governance model that integrates payment rules, clinical indications, service quality, patient experience, and equity monitoring [5].

### 4.6. Data governance, cybersecurity, and AI regulation

Internet healthcare depends on the secure circulation of consultation records, electronic prescriptions, medical images, remote-monitoring data, insurance information, and personal health information across hospitals, platforms, pharmacies, insurers, and patients. Evidence from China's electronic medical record integration experience shows that fragmented administrative authority, non-uniform information standards, institutional reluctance to share data, and patient authorization issues can hinder medical data integration [31]. The 2018 trial measures required internet diagnoses and treatment systems to support identity verification, electronic medical records, information security, and traceable service processes [5]. The 2022 supervision rules further strengthened online-offline regulatory linkage by incorporating internet diagnoses and treatments into supervision through provincial regulatory platforms [28].

**Table 1.** Minimum quality and safety requirements for internet diagnosis and treatment in Chinese public hospitals.

Domain	Minimum requirement
Service scope	Online services should mainly cover follow-up for selected common and chronic diseases and family-doctor contract services.
First diagnosis and acute conditions	First diagnosis, acute symptoms, unstable conditions, and cases requiring physical examination should be referred to offline care.
Institutional accountability	Internet diagnosis and treatment should rely on licensed medical institutions or internet hospitals linked to physical institutions.
Identity and medical records	Real-name authentication, prior medical-record review, electronic records, and traceable consultation records should be required.
Prescription safety	E-prescriptions should be reviewed, traceable, and linked to safe drug dispensing or delivery pathways.
Online-offline linkage	Online follow-up should include offline referral or reassessment when symptoms, test results, or risk status change.
Supervision	Internet diagnosis and treatment should be incorporated into hospital quality-control systems and provincial regulatory platforms.
Data protection and consent	Platforms should ensure data security, personal-information protection, informed consent, and physician accountability.

Privacy and consent remain important concerns. A 2024 content analysis of internet hospital apps in mainland China found variable compliance with privacy-policy requirements and recommended stronger informed consent and personal-information protection [32]. AI-assisted triage and decision support should be interpreted as emerging supportive functions rather than mature substitutes for professional clinical judgment. Yang et al. described the development and application of an AI-enabled outpatient triage system under the “Internet Plus Health Care” model, thereby illustrating the potential of AI to improve prehospital guidance and workflow efficiency [33]. However, policy discussions should avoid portraying AI as a fully mature or autonomous diagnostic system, because the responsibility for diagnoses, prescriptions, referrals, and patient safety remains with licensed physicians and accountable medical institutions. Additionally, physician interview evidence suggests that internet-hospital care requires clearer patient education, informed consent, communication standards, and realistic expectations about what online services can and cannot provide [27]. Therefore, data governance and AI regulation are core components of high-quality internet healthcare, and the minimum quality and safety requirements are summarized in Table 1.

## 5. Institutional practices, innovations, and clinical evidence

Chinese public hospitals have not implemented internet healthcare as a single digital product, but rather as a set of institutionally governed service pathways that connect online consultations, offline clinical accountability, remote monitoring, electronic prescriptions, insurance settlements, inter-hospital collaborations, and patient follow-ups. Evidence suggests that this hospital-centered model is most clinically defensible when used as an adjunct to established care pathways, especially for stable chronic disease follow-ups, medication renewals, remote monitoring, patient education, and secondary prevention—rather than as a replacement for the first diagnosis, emergency care, physical examination, or complex diagnostic decision-making.

### 5.1. Clinical outcomes and patient safety: what has been demonstrated?

Emerging empirical evidence suggests that internet healthcare and related digital health interventions can improve selected chronic disease outcomes when embedded in structured monitoring and follow-up systems [34]. In a real-world analysis of 2883 patients with diabetes managed through Tianjin's digital integrated health platform, participation in digital chronic disease management was associated with reductions in fasting glucose, postprandial glucose, and HbA1c, with larger improvements among highly adherent patients [35]. Similarly, community-based evidence from Beijing suggested that intelligent chronic disease management improved the follow-up regularity and glycemic control among patients with type 2 diabetes [36]. Additionally, randomized evidence supports the telemedicine-assisted structured self-monitoring of blood glucose, which improved the glycemic control and reduced the hypoglycemia risk compared with traditional glucose monitoring [37].

Hypertension and cardiovascular secondary prevention provide additional outcome-level evidence. A randomized controlled trial in Ningxia found that an mHealth app-based intervention improved the six-month blood pressure control among patients with hypertension, with control rates of 90.1% in the intervention group and 65.2% in the usual-care group [38]. In patients with coronary heart disease after percutaneous coronary intervention, a web-based telemedical interventional management system reduced one-year major adverse cardiac and cerebrovascular events from 5.3% to 3.5%, while improving the blood-pressure control and medication adherence [39].

Moreover, evidence on access and service burden supports selected online follow-up pathways. A mixed-methods study of 18,473 patients and 39,239 online regular follow-up visits at an internet hospital found that online follow-ups reduced the travel time by 1–9 hours and transportation costs by ¥6–¥991, with greater savings among patients living farther from the hospital. Importantly, this benefit was achieved through safeguards such as patient selection, medical-record review, prior offline diagnoses, and recommendations of offline reassessments when needed [5].

However, the diagnostic accuracy and patient safety evidence remains mixed. Standardized-patient studies have found variations in checklist completion, diagnostic quality, prescribing, patient-centeredness, timeliness, and cost across telemedicine platforms [6],[40]. In postpartum depression consultations, asynchronous webchat consultations were inferior to in-person consultations in diagnostic accuracy, guideline adherence, and patient-centeredness [7]. These findings suggest that internet hospitals should be positioned as safe and useful adjuncts for selected follow-up and chronic-care functions, rather than as replacements for the first diagnosis, emergency care, mental-health risk assessment, unstable conditions, or cases requiring physical examination.

### 5.2. Definition and organizational models of internet hospitals

In this review, an internet hospital refers to a regulated digital medical-service entity legally anchored in a licensed physical medical institution. Under China's 2018 regulatory framework, internet hospitals may either operate as the “second name” of a physical medical institution or as an independently established internet hospital that relies on a licensed physical institution for clinical accountability. This institutional anchoring links online services to physician credentialing, medical-record access, prescription review, quality control, data security, and offline referral pathways [3].

Empirical studies show that public hospitals, especially tertiary public hospitals, have been central actors in China's internet-hospital development. A documentary and qualitative interview study identified 268 licensed internet hospitals, including 153 public internet hospitals, and found that public tertiary hospitals played a leading role in serving patients with common diseases, chronic diseases, and access barriers in remote or rural areas [17]. Earlier cross-sectional evidence showed that, by March 2017, 68 internet hospitals were identified in mainland China, but many remained immature because of scarce online physician availability, incomplete insurance coverage, and an uneven service capacity [1]. These findings suggest that integration with physical hospitals was not only a regulatory requirement, but also a practical response to early weaknesses in the platform capacity, physician supply, medical-record access, and reimbursement.

In practice, China's internet hospitals can be grouped into four broad models: public-hospital-integrated models, hospital-affiliated platform models, regional or government-coordinated platforms, and enterprise-led but institution-dependent models [41]. The First Affiliated Hospital, Zhejiang University School of Medicine, illustrates the physical-hospital-based model, in which online consultations, electronic prescriptions, medication delivery, quality control, and network-security governance are incorporated into the hospital's management structure [42]. Therefore, the organizational innovation of Chinese internet hospitals lies less in replacing hospitals with virtual platforms than in extending public-hospital functions into regulated online-offline service pathways.

### 5.3. Integrated service pathways: patient-facing and inter-institutional care

Patient-facing internet-hospital services usually begin before clinical encounters. Public hospitals use mobile apps, WeChat mini-programs, and web portals for appointment booking, triage, payments, report retrieval, and patient instructions. These functions may reduce the administrative burden and improve the patient flow, but their clinical value depends on whether they are integrated with offline care pathways and medical records rather than functioning as isolated convenience tools.

Online follow-ups are the most mature patient-facing clinical pathway. For stable patients with a prior offline diagnosis, internet hospitals can provide follow-up consultations, medication renewals, test-result reviews, chronic disease education, risk stratification, and offline referrals when necessary. The Zhejiang University case shows how these services can be operationalized through record review, physician consultation, e-prescription, medication delivery, quality control, and network-security arrangements within a physical hospital [42]. During COVID-19, Peking Union Medical College Hospital used internet technologies to support remote consultation, information management, and continuity of care under infection-control pressure, thereby illustrating how leading tertiary hospitals can rapidly mobilize digital tools during public-health emergencies [43]. A national cross-sectional study similarly documented that many internet hospitals provided online consultations, prescriptions, drug delivery, and medical-insurance-related services during the pandemic [21].

Internet healthcare in public hospitals also includes physician-to-physician and hospital-to-hospital telemedicine. This pathway includes remote specialist consultation, remote imaging, remote pathology, remote electrocardiography, tele-ICU consultation, emergency guidance, multidisciplinary case discussions, and cross-regional referrals. Its public-health value lies in extending specialist expertise from tertiary hospitals to county hospitals, community health centers, and resource-limited regions. Evidence from China suggests that telemedicine has increasingly been used to address the unequal distribution of healthcare resources by linking higher-level hospitals with lower-level institutions [15],[44]. During the early COVID-19 response, online health-service projects showed that physicians were the main workforce, while nurses, pharmacists, and other professionals also participated; however, the regional distribution remained uneven and primary-care participation was limited [45].

For high-acuity settings such as ICU or emergency care, internet healthcare should be framed as specialist support rather than as a replacement for bedside care. Tele-ICU and emergency teleconsultation can help tertiary specialists guide lower-level hospitals on triage, imaging interpretation, treatment planning, and transfer decisions, but they require standardized escalation protocols, timely data transmission, and clear accountability.

### 5.4. Technology-enabled hospital integration: EMR, AI, 5G, and remote monitoring

Technology-enabled integration is a defining feature of internet healthcare in Chinese public hospitals, but the maturity of different technologies varies. Electronic medical records and hospital information systems provide the foundation for safe online follow-ups because physicians must be able to review prior diagnoses, medications, test results, imaging, and offline visits before issuing advice or prescriptions. However, electronic medical record integration remains constrained by fragmented administrative authority, non-uniform information standards, institutional reluctance to share data, and patient authorization issues [31].

AI-assisted tools should be described as emerging support functions rather than mature autonomous diagnostic systems. A 2024 study described the development and application of an AI-enabled outpatient triage system under the “Internet Plus Health Care” model, thereby illustrating how AI may improve the prehospital guidance and workflow efficiency [33]. Nevertheless, AI triage and decision support should remain under physician supervision because the responsibility for diagnoses, prescriptions, referrals, and patient safety remains with licensed professionals and accountable medical institutions.

5G-enabled remote monitoring and hospital-at-home models represent another frontier. A smart home ward-based hospital-at-home model in China showed how 5G-enabled monitoring, mobile communication, and hospital coordination can extend selected hospital-level services into the home setting [46]. These models may be useful for post-discharge monitoring, heart failure management, stroke recovery, rehabilitation, and chronic disease follow-ups, but they require careful patient selection, device reliability, escalation pathways, data security, and sustainable staffing models.

### 5.5. Hospital-centred internet healthcare in China and international comparison

China's internet healthcare model should be interpreted in relation to its hospital-centered health-system structure. In many health systems, telemedicine is embedded in primary-care or family-doctor pathways, where the general practitioner serves as the first point of contact, care coordinator, and gatekeeper for specialist referrals. By contrast, Chinese patients often have greater freedom to directly seek care from large urban hospitals, while high-quality specialists, diagnostic technologies, and public trust remain concentrated in tertiary public hospitals. A hypertension mHealth trial noted that many Chinese patients seek treatments in larger urban hospitals rather than local primary-care clinics because the hierarchical healthcare system is less strict and patients have more freedom to choose where to receive treatment [38].

This difference explains why Chinese internet hospitals often function as hospital-centered digital extensions rather than primary-care-centered telemedicine platforms. The advantage is that they can rapidly extend scarce specialist resources, support cross-regional consultations, and provide follow-up for patients who would otherwise repeatedly return to tertiary hospitals. The risk is that hospital-centered digital expansion may increase demand for large public hospitals and reinforce dependence on tertiary institutions if online services are not integrated with family-doctor teams, community health service centers, and county medical communities [47]. Therefore, the future value of China's model not only depends on the platform capacity, but also on whether tertiary internet hospitals can support, rather than bypass, primary-care continuity and hierarchical diagnoses and treatments.

## 6. Challenges and barriers to high-quality integration

### 6.1. The evidence-safety paradox and regulatory heterogeneity

The first barrier is an evidence-safety paradox: internet hospitals are being institutionalized as routine infrastructure, but their strongest evidence remains confined to selected, lower-risk service scenarios. Digital chronic disease management has shown benefits for diabetes control, including improvements in glycemic indicators and HbA1c, and telemedicine-assisted glucose self-monitoring has further supported diabetes self-management [35]–[37]. Additionally, randomized and interventional evidence supports online or mobile follow-ups for hypertension and post-percutaneous coronary intervention (PCI) cardiovascular secondary prevention, including better blood-pressure control, medication adherence, and reduced major adverse cardiovascular and cerebrovascular events [38],[39]. Moreover, online regular follow-up can reduce the travel time and transportation costs when embedded in prior offline diagnoses, medical-record reviews, and escalation safeguards [5]. However, these findings cannot be generalized to the first diagnosis, emergency care, complex multimorbidity, acute symptom triage, mental-health risk assessment, or conditions which require physical examinations. Standardized-patient studies show variations in the diagnostic quality, guideline adherence, prescribing appropriateness, and patient-centeredness, while asynchronous webchats performed worse than in-person consultations for postpartum depression diagnoses and management [6],[7],[40].

National rules anchor internet diagnoses and treatments in licensed physical medical institutions and require online services to be incorporated into provincial supervision systems [3],[28]. The underlying contradiction is Decentralized Management vs. Centralized Accountability: provinces and institutions locally manage platforms, whereas the clinical responsibility remains concentrated in the licensed physician and hosting hospital. When delayed diagnoses, unsafe prescribing, failed referrals, or medication-delivery errors occur, the responsibility may be difficult to allocate among the internet platform, physical hospital, physician, pharmacy, insurer, and patient's local institution. Therefore, high-quality integration requires a shift from platform approval and service volume toward outcome-based indicators, including the diagnostic concordance, escalation appropriateness, prescription safety, adverse-event reporting, hospitalization after online consultation, continuity of care, patient-reported outcomes, and equity-stratified access.

### 6.2. Organizational resilience and inclusive change leadership

The second barrier is organizational inertia. Internet healthcare is often introduced as a technical upgrade, yet its success depends on work redesign, leadership behaviors, professional motivation, and institutional learning. Internet hospitals are not simply IT procurement projects. They alter the work of clinical, administrative, quality-control, insurance, and IT teams. If online services are added to already crowded outpatient systems without protected time, fair compensation, clear clinical boundaries, or team-based workflows, then digital transformation becomes additional labor rather than service innovation. This is especially problematic in public hospitals, where clinicians may be expected to absorb online consultations, e-prescription review, remote communications, and patient follow-ups without corresponding changes in workload accounting or performance evaluations.

Although not specific to Chinese internet hospitals, organizational-change literature provides a useful lens to understand this bottleneck. Evidence-based human-resource management research indicates that high-involvement work practices can promote nurses' innovative work behavior through job crafting and supportive leadership [48]. For internet hospitals, this implies that clinicians and nurses should not be treated as passive end users of digital platforms. They should be engaged as co-designers of triage rules, escalation pathways, remote-monitoring workflows, prescription-review processes, patient-education scripts, and safety indicators. Without such involvement, the platform design may reproduce institutional inertia: technology changes, but clinical workflows, accountability structures, and incentive systems remain unchanged.

Inclusive leadership is equally central to high-quality integration. Organizational-change research suggests that inclusive leadership may promote innovative work behavior through psychological empowerment [49]. In internet hospitals, psychological empowerment means that frontline staff feel able to report unsafe workflow designs, suggest triage modifications, challenge unrealistic platform functions, and participate in service redesign without fear of blame. This is not merely a managerial preference. It is a patient-safety condition, because online care creates new areas of uncertainty, including incomplete physical examinations, text-based ambiguity, delayed responses, patient complaints, and unclear responsibility for online errors. Inclusive leadership can convert these uncertainties into learning opportunities rather than sources of defensive practice.

Leadership quality also matters because internet healthcare increases emotional and communicative labor. The Leadership & Organization Development Journal (LODJ) study links leadership, emotional intelligence, job satisfaction, and physician performance, a relationship that is directly relevant to online clinical work [50]. Physicians working in internet hospitals must manage patient expectations, doctor-patient communication, trust, and decisions about when online care is no longer sufficient [27],[51]. If these tasks remain invisible in staffing models, then physicians may perceive online services as medicolegal exposure rather than professional innovation.

Dynamic capabilities explain why similar digital investments may produce different institutional outcomes. Public-hospital research suggests that employee dynamic capabilities are associated with innovative work behaviors and job performances [52]. A related study further indicates that technological readiness affects performance through digital health transformation and innovative work behaviors [53]. For Chinese public hospitals, the decisive capability is not purchasing platforms, but sensing clinical demand, redesigning workflows, training multidisciplinary teams, reallocating time, learning from errors, and adapting online-offline pathways. Therefore, organizational resilience requires clinician-led workflow redesign, protected online service time, fair compensation, digital training, leadership support, multidisciplinary internet-hospital teams, and clearly defined responsibility boundaries.

### 6.3. Infrastructure interoperability and algorithmic governance

The third barrier is infrastructural fragmentation. Internet healthcare promises continuity, yet safe online follow-ups depend on access to prior diagnoses, prescriptions, laboratory results, imaging, procedures, allergies, offline visits, and referral histories. In China, electronic medical-record integration remains constrained by fragmented administrative authority, non-uniform data standards, institutional reluctance to share data, and patient authorization barriers [31]. Again, this reflects Decentralized Management vs. Centralized Accountability: institutions and provinces control data systems, reimbursement interfaces, and regulatory platforms, but the online physician remains clinically accountable for decisions made with incomplete information.

AI-assisted triage intensifies this governance challenge. AI-enabled outpatient triage may improve the prehospital guidance and workflow efficiency [33]. However, algorithms may misclassify symptoms, miss atypical presentations, reproduce training-data biases, or falsely reassure patients who require urgent offline care. If AI recommendations, physician decisions, platform prompts, and hospital supervision are not linked through auditable decision logs, then the responsibility for AI-assisted errors becomes difficult to trace. Therefore, cybersecurity, privacy protection, and data governance should be treated as patient-safety and accountability issues rather than purely technical afterthoughts [54]. Internet hospitals rely on identity verification, e-prescriptions, online payments, insurance settlements, drug delivery, and the storage of sensitive health data; privacy-policy compliance among internet hospital apps remains variable in mainland China [32]. Lower-resource institutions may lack regular cybersecurity audits, disaster-recovery systems, and real-time monitoring capacity. Therefore, high-quality integration requires interoperable data standards, cross-institution record exchanges, algorithmic accountability, AI performance monitoring, cybersecurity audits, and explicit legal responsibility for AI-assisted decisions.

### 6.4. Socio-demographic exclusion and global transferability

The fourth barrier is structural exclusion. Internet healthcare is designed to improve access, but it may exclude patients who most need convenient care. Older adults, rural residents, people with low education, patients with disabilities, and those without family support may face a digital literacy trap [27],[55],[56]. The problem is not simply smartphone use. The internet-hospital pathway increasingly requires real-name authentication, account registration, appointment selection, symptom descriptions, online payments, insurance verification, electronic prescriptions, drug-delivery confirmation, report retrieval, and follow-up communications. A patient may fail at any step, even when the clinical service exists. Recent evidence shows that eHealth adoption differs by age, residence, education, income, chronic disease status, eHealth literacy, perceived usefulness, and perceived ease of use [55]. Additionally, health-app benefits may be greater among people with higher consumption levels, a higher-tier city residence, and a higher digital literacy, thus suggesting that digital health can reproduce socioeconomic gradients [57].

**Figure 2.**
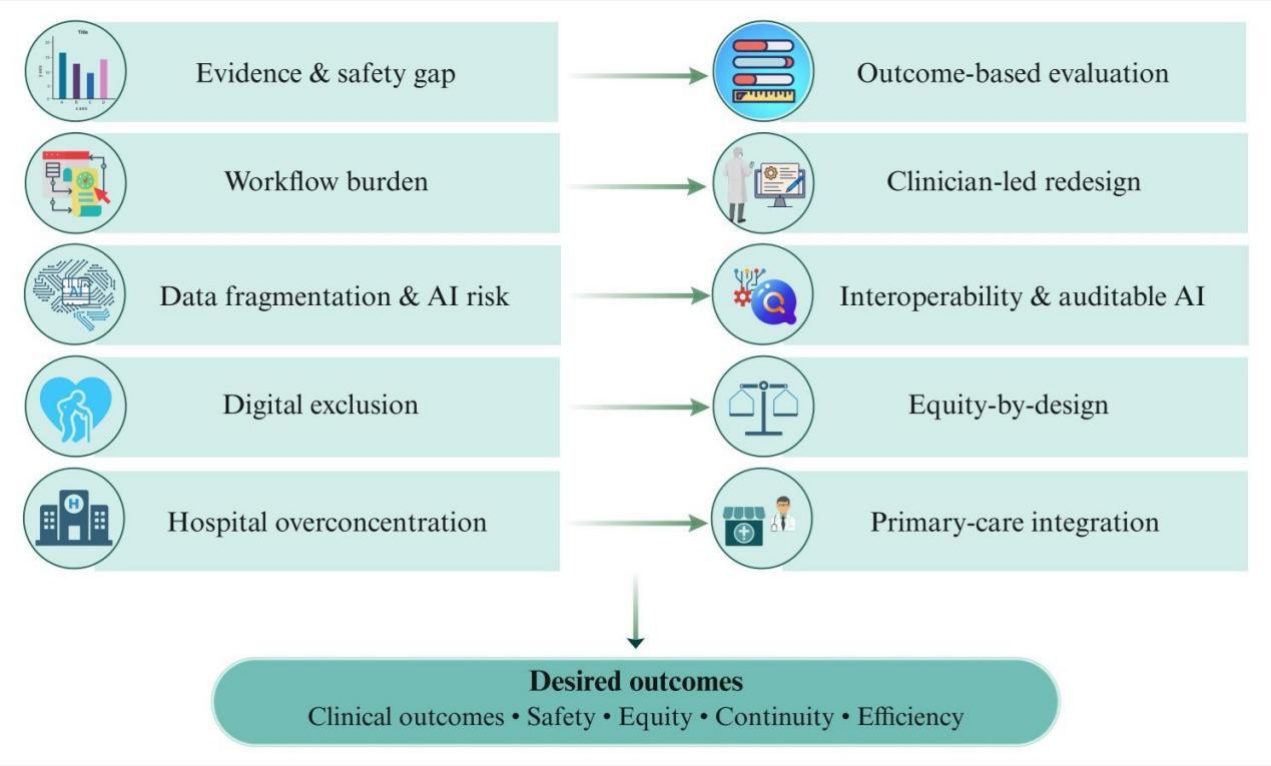
Challenge-to-strategy mapping for high-quality internet healthcare in Chinese public hospitals.

Therefore, solutions must be social-engineering interventions, not only patient education. Public hospitals should retain offline windows, telephone support, elderly-friendly interfaces, family-assisted accounts with consent controls, community digital navigators, and primary-care support for online follow-ups. Additionally, regional inequity limits integration because broadband stability, device access, trained staff, and the local implementation capacity remain uneven outside large urban hospitals. China's hospital-centered model may be the most transferable to countries with large public hospitals, specialist concentration, regional disparities, and rapid digital infrastructure development, but less transferable to systems organized around strong general physician (GP) gatekeeping or decentralized private providers. Finally, this review has an evidence-base limitation: some early milestones, policies, and institutional practices are available only in Chinese or gray literature. These sources were used mainly for historical and policy contexts, whereas peer-reviewed evidence was prioritized for clinical outcomes, diagnostic quality, safety, and service effectiveness. The relationships between these barriers and the corresponding strategic responses are summarized in Figure 2.

This diagram maps the main barriers to high-quality internet healthcare in Chinese public hospitals to corresponding strategic responses. The key challenges include the evidence and safety gap, workflow burden, data fragmentation and AI-related risk, digital exclusion, and hospital overconcentration. The corresponding responses include outcome-based evaluations, clinician-led workflow redesigns, interoperability and auditable AI, equity-by-design, and primary-care integration. The desired outcomes are improved clinical outcomes, patient safety, equity, continuity of care, and service efficiency. This framework reflects the need to move beyond platform expansion toward outcome-oriented, safe, equitable, and integrated internet healthcare.

## 7. Future outlook and strategic recommendations

### 7.1. From volume to value: establishing outcome-based quality governance

The next stage of internet healthcare in Chinese public hospitals should move from platform expansion to outcome-based quality governance. Evaluation should no longer focus on the number of licensed internet hospitals, online consultations, or e-prescriptions, but rather on whether digital services improve outcomes, safety, continuity, equity, and efficiency. HbA1c control is appropriate for diabetes management because digital platforms and telemedicine-assisted glucose monitoring improved diabetes outcomes [35],[37]. Blood-pressure control should be included because mHealth improved hypertension management [38]. Medication adherence and major adverse cardiovascular and cerebrovascular events should be monitored in post-PCI care because web-based telemedical management improved secondary prevention [39]. Diagnostic concordance, guideline adherence, prescribing appropriateness, and escalation timeliness should be assessed because standardized-patient studies found variable online diagnostic quality [6],[40].

A unified safety incident reporting system is essential. Reportable events should include delayed diagnoses, unsafe prescribing, failed referrals, medication-delivery errors, privacy breaches, AI-triage errors, and platform outages. The 2018 trial measures and 2022 supervision rules provide the basis for institutional accountability and provincial supervision. Privacy-policy compliance should be included because internet hospital apps show variable information-protection practices [32]. AI incidents should be auditable because AI triage is increasingly used for prehospital guidance [33].

### 7.2. System integration: empowering the hierarchical care model

Internet healthcare should become a tool for system integration rather than unlimited tertiary-hospital traffic expansion. China's hospital-centered model can mobilize a specialist's expertise but may reinforce the siphon effect of large urban hospitals if online platforms bypass county hospitals, community health service centers, and family-doctor teams. Digital platforms should support hierarchical diagnoses and treatments by positioning tertiary hospitals as specialist hubs while strengthening county and community institutions as providers of first-contact care, follow-ups, and continuity management.

Remote consultation, imaging, pathology, electrocardiogram (ECG) interpretation, tele-ICU support, emergency guidance, and multidisciplinary discussion should be embedded into county medical communities because telemedicine helps address unequal healthcare-resource distribution in China [15]. Early COVID-19 eHealth experience showed rapid physician-led online service expansion but uneven regional distribution and limited primary-care participation [45]. AI-assisted triage may guide patients to appropriate service levels but should remain embedded in accountable clinical pathways rather than used as an autonomous gatekeeper [33].

### 7.3. Equity-by-Design: bridging the multi-dimensional digital divide

Digital health equity should be a design principle, not a downstream compensation strategy. Older adults, rural residents, people with low education, patients with disabilities, and those without family support may face exclusion across authentication, appointment booking, symptom entry, online payments, insurance verification, e-prescriptions, drug delivery, and report retrieval. Physician interviews identified access barriers among older, rural, and digitally disadvantaged patients [27]. Survey evidence shows that eHealth adoption differs by age, residence, education, income, chronic disease status, eHealth literacy, perceived usefulness, and perceived ease of use [55]. Mobile health benefits may be greater among people with higher consumption levels, a higher-tier city residence, and a higher digital literacy [57].

Future platforms should adopt an inclusive design, including simplified registration, large-font and voice-guided interfaces, telephone-assisted consultations, family-assisted accounts with consent controls, offline windows, pharmacy-based support, and community digital navigators. Digital equity indicators, such as age-stratified access, rural-urban use, disability accessibility, failed-login rates, incomplete consultation rates, and offline fallback availability, should be incorporated into high-quality development assessments. Future research should use real-world evidence, prospective cohorts, standardized-patient studies, implementation research, and multicenter trials to evaluate the long-term effectiveness, safety, equity, and transferability.

## 8. Conclusions

Over the past four decades, internet healthcare in Chinese public hospitals has evolved from early telemedicine experiments into a regulated online–offline service model embedded in public-hospital reform. Supported by policy stewardship, technological development, and hospital-level implementation, China has developed a distinctive hospital-centered approach to digital healthcare. Current evidence suggests that this model may improve selected aspects of chronic disease follow-ups, medication adherence, cardiovascular secondary prevention, and patient travel burden, but its effects on the diagnostic accuracy, adverse events, emergency escalation, and long-term outcomes remain insufficiently established.

Therefore, future high-quality developments should move beyond platform expansion toward outcome-based evaluations, patient-safety governance, interoperable data systems, organizational capacity building, and equity-by-design. China's experience may offer useful lessons for health systems with large public hospitals and regional disparities, but its transferability depends on the local regulatory capacity, digital infrastructure, primary-care organization, and clinical accountability mechanisms.

## Use of AI tools declaration

The authors acknowledge the use of DeepSeek-V3.1 (DeepSeek-AI, Hangzhou, China) solely for English-language polishing, grammar correction, and readability improvement. The tool was not used for literature searching, data extraction, or generation of scientific content. All AI-assisted edits were reviewed, verified, and approved by the authors, who take full responsibility for the accuracy and integrity of the manuscript.
